# Genome-Resolved Characterization of Structure and Potential Functions of the Zebrafish Stool Microbiome

**DOI:** 10.3389/fcimb.2022.910766

**Published:** 2022-06-15

**Authors:** Masood ur Rehman Kayani, Syed Shujaat Ali Zaidi, Ru Feng, Kan Yu, Yushu Qiu, Xiaogang Yu, Lei Chen, Lisu Huang

**Affiliations:** ^1^ Department of Infectious Diseases, Xinhua Children’s Hospital, Xinhua Hospital, Shanghai Jiao Tong University School of Medicine, Shanghai, China; ^2^ Department of Immunobiology, University of Arizona, Tucson, AZ, United States; ^3^ Shanghai Institute of Immunology, Shanghai Jiao Tong University School of Medicine, Shanghai, China; ^4^ School of Life Sciences, Fudan University, Shanghai, China; ^5^ Ministry of Education and Shanghai Key Laboratory of Children’s Environmental Health, Xinhua Hospital, Shanghai Jiao Tong University School of Medicine, Shanghai, China

**Keywords:** genome-resolved metagenomics, metagenome-assembled genomes, metabolic potential, comparative genomics,*Paucibacter*, zebrafish stool microbiome

## Abstract

Zebrafish have been used as a model organism for more than 50 years and are considered an excellent model for studying host-microbiome interactions. However, this largely depends on our understanding of the zebrafish gut microbiome itself. Despite advances in sequencing and data analysis methods, the zebrafish gut microbiome remains highly understudied. This study performed the *de novo* metagenome assembly and recovery of the metagenome-assembled genomes (MAGs) through genome binning (and refinement) of the contigs assembled from the zebrafish stool. The results indicate that majority of the MAGs had excellent quality i.e. high completeness (≥90%) and low contamination levels (≤5%). MAGs mainly belong to the taxa that are known to be members of the core zebrafish stool microbiome, including the phylum Proteobacteria, Fusobacteriota, and Actinobacteriota. However, most of the MAGs remained unclassified at the species level and reflected previously unexplored microbial taxa and their potential novelty. These MAGs also contained genes with predicted functions associated with diverse metabolic pathways that included carbohydrate, amino acid, and lipid metabolism pathways. Lastly, we performed a comparative analysis of *Paucibacter* MAGs and reference genomes that highlighted the presence of novel *Paucibacter* species and enriched metabolic potential in the recovered MAGs.

## Introduction

The zebrafish (*Danio rerio*), a freshwater teleost fish (Engeszer et al., 2007), was established as a model organism in the late 1960s by George Streisinger for studying complex vertebrate biology (Burns and Guillemin, 2017). Since then, zebrafish have been extensively used for studying diverse topics in research which include, but are not limited to, developmental biology (Roper and Tanguay, 2018), cancers (Amatruda et al., 2002), and ecotoxicology (Nagel, 2002). The ease of its management drives the success of zebrafish as a model organism in laboratories, rapid development, high fecundity, and low handling cost in contrast with other models (Stagaman et al., 2020). These features also enable us to develop newer zebrafish models to study previously unexplored biological and biomedical research avenues.

The microbiome is essential for maintaining the host’s health and homeostasis, yet our understanding of these mechanisms remains limited (Gilbert et al., 2018). In recent years, zebrafish has emerged as a powerful model for elucidating different host-microbiome interactions (Rawls et al., 2004; Bates et al., 2006; Rawls et al., 2006; Bates et al., 2007; Cheesman et al., 2011). However, the success and large-scale application of zebrafish as a model to study complex host-microbiome interactions and explain the underlying molecular mechanisms massively depend on thorough knowledge regarding the diversity and functional potential of the zebrafish stool microbiome. Initial efforts to characterize the zebrafish stool microbiome involved using the 16S rRNA sequencing of the stool (Rawls et al., 2004; Rawls et al., 2006; Brugman et al., 2009; Roeselers et al., 2011), which has several limitations. Whole metagenome shotgun sequencing (MGS)-based surveys of zebrafish stool microbiome can provide more accurate and descriptive insights into the taxonomic diversity and functional potential. In a recent effort, Kayani et al., 2021b exposed the zebrafish to different environmental concentrations (much lower than therapeutic concentrations) of ﻿oxytetracycline and sulfamethoxazole from the larval stage to adulthood (~120 days). They highlighted the differences between control and exposed groups using MGS (Kayani et al., 2021b). In addition, Gaulke et al. generated a set of ~1.5 M non-redundant genes from 29 individual zebrafish using MGS and RNAseq to understand the zebrafish gut microbiome diversity. However, their work is currently unpublished and only available as a preprint (Gaulke et al., 2020). Albeit, we believe that presently the scale of application of MGS in understanding the zebrafish-related research is not large enough and demands more effort.

Recent advances in sequencing technologies and improvements in computational methods have introduced a new dimension in metagenome data analysis, i.e., genome-resolved metagenomics. These genome-resolved metagenome analyses usually involve *de novo* metagenome assembly and subsequent recovery of microbial population genomes, also termed metagenome-assembled genomes (MAGs), from the assemblage (Kayani et al., 2021a). Using this approach, microbial population genomes have been recovered from various environmental settings, especially human gut microbiome (Nielsen et al., 2014). These studies have also contributed to identifying the first genomic representatives of certain uncultivable microbes and novel insights into the metabolic potential of microbes (Mukherjee et al., 2017; Parks et al., 2017). Therefore, genome-resolved metagenome analysis provides a better route for a comprehensive understanding of microbial diversity, adaptations, and their correlation with the hosts. In the case of zebrafish, no genome-resolved analyses have been published to the best of our knowledge. Furthermore, representative microbial genomes recovered from the zebrafish stool are non-existent in the reference genome repositories.

This study cataloged the zebrafish stool microbiome’s microbial diversity and functional potential using genome-resolved metagenome analysis. Our primary objectives were to (i) recover microbial-population genomes of the draft and high-quality from the zebrafish stool microbiome, (ii) identify the most prevalent and previously unobserved taxa from the zebrafish stool microbiome, (iii) elucidate the metabolic potential of the recovered MAGs through functional annotation, and (iv) perform a comparative analysis between MAGs recovered from the zebrafish stool microbiome and reference genomes for characterizing their similarities and differences. We recovered MAGs from the zebrafish stool microbiome which were mainly high-quality and exhibited a certain degree of novelty. These MAGs would be essential for performing comparative analysis between zebrafish and human gut microbiomes (or gut microbiome of other model organisms) and assist in better exploitation of zebrafish as a model to study the gut microbiome in the future.

## Materials and Methods

### Zebrafish Husbandry and Maintenance

Wild-type zebrafish (AB strain) was purchased from the China Zebrafish Resource Center (Wuhan, China) and reared at Xinhua Hospital (affiliated to Shanghai Jiao Tong University School of Medicine, Shanghai). Three male and two female adult zebrafish were placed in a breeding tank, separated overnight using a baffle before the experiment to obtain embryos. The baffle was removed the following day to allow zebrafish to mate. Viable embryos were collected within 20 minutes of fertilization, while dead embryos were discarded. This experiment was performed in four different batches at different time points (**Supplementary Table 1**). Embryos were maintained in an incubator at a constant temperature of 28 ± 0.5°C and supplied with paramecium feed twice a day after hatching. After two weeks, zebrafish were transferred to different tanks (2-20L) under a 12:12 h light: dark photoperiod and fed twice a day with brine shrimp. The average number was 5-20 zebrafish per tank whereas their age ranged between 4-8 months. Daily water changes were performed with clean, fresh, de-chlorinated water. The water temperature and pH were maintained at 28 ± 0.5°C and 6.8-7.5, respectively.

### DNA Extraction and Metagenomic Sequencing

The zebrafish stool samples from adult zebrafish were collected separately from all five tanks as described elsewhere (Gaulke et al., 2019). Briefly, a group of ~5-10 zebrafish, randomly selected from the main tanks, were separated into a smaller tank with clean water (maintained at the same temperature and pH as the main tank) prior to sample collection. Stool samples were collected using a new 5-ml sterile transfer pipet and frozen at -20°C until used. Metagenomic DNA was extracted following a previously established protocol (Gilchrist et al., 2007) using Qiagen PowerFecal Kit (Catalog No. 12830–50; Qiagen, Hilden, Germany). Briefly, 150 mg of fecal pellets were homogenized in PowerBead Tubes using 750 μL of bead solution, followed by Solution C1. The samples were then briefly vortexed and incubated at 65°C for 10 min. PowerBead Tubes were horizontally attached to a vortex mixer using the MO BIO Vortex Adapter for enhanced homogenization (Catalog No. 13000-V1-24; Scientific Industries, Bohemia, NY) and shaken for 15 min. Subsequently, centrifugation was performed for 30 sec at 10,000 g, and the supernatant was collected in 2 mL collection tubes. All samples were eluted in 100 μL of Solution C6 and air-dried at room temperature for 15 min. Final centrifugation was performed, and the extracted DNA was transferred to ThermoFisher Matrix 500 μL screw-top tubes and stored at -20°C until further usage.

500 ng of DNA was used to construct the paired-end metagenomic libraries with the Nextera DNA Flex Library Prep kit (Illumina, CA, US). The manufacturer’s protocol was used for the library preparation, which briefly included fragmentation and adapter ligation of the DNA, polymerization of the adapter-ligated library, and purification of the amplified library. Metagenomic sequencing of the purified library was performed using the Illumina Hiseq X Ten (Illumina, CA, US) sequencer to generate paired-end (PE) reads with approximately 150 bp per read-end. In addition to these five zebrafish metagenomes, we included three metagenomes from our previous study (Kayani et al., 2021b) in the current analysis.

### Read Quality Control and Metagenome Assembly

Raw metagenomic reads were preprocessed for the removal of low-quality sequences (quality lower than Q20), adapter sequences, and ambiguous bases (N) using FastQC (v0.11.8) and TrimGalore (v0.5.0) (Andrews, 2010; Krueger, 2015). Furthermore, reads were mapped to the zebrafish reference genome (GCA_000002035.4) and the Human genome (hg38) using BMTagger (v1.1.0) (Rotmistrovsky and Agarwala, 2011), and successfully mapped reads were removed from downstream analyses.

Metagenomic *de novo* assembly was performed for the high-quality reads using MEGAHIT assembler (v1.1.4) (Li et al., 2015) with *k* -mers ranging from 29 to 101 (–k-min 29, –k-max 101), a *k*-mer step size of 10 (–k-step 10) and minimum contig length of 1000 bp (–min-contig-len 1000). Since the study’s primary goal was the recovery of microbial genomes, we used a minimum contig length of 1000 bp, as required by most of the binning tools. Each metagenome sample was initially assembled individually to allow assembly of high coverage contigs. Therefore, to further assemble additional (low coverage or less abundant) contigs, we performed an additional co-assembly of all the zebrafish stool metagenome samples using the aforementioned parameters. High-quality reads were then mapped to corresponding per-sample assemblies and co-assembly using the ultrafast and memory-efficient alignment tool, i.e., Bowtie2 (v2.3.5) (Langmead and Salzberg, 2012). The alignment was sorted using the *sort* function in the SAMtools (v1.9) (Li et al., 2009) and further used for calculating the depth of each contig in both types of assemblies with the *jgi_summarize_bam_contig_depths* utility provided with the MetaBAT tool (v2.12.1) (Kang et al., 2015).

### Recovery of Metagenome-Assembled Genomes

For the recovery of MAGs, we used a computationally exhaustive, multi-tool genome binning and refinement-based approach. The initial binning step involved the application of two different genome binning tools, i.e., MetaBAT2 (v2.12.1) (Kang et al., 2019) and MaxBin2 (v2.2.6) (Wu et al., 2016). Binning with MetaBAT2 involved the usage of the contig depths, calculated as mentioned in the previous section, and a minimum contig length of 1500 (option: ‘-m 1500’), whereas MaxBin2 was used with default parameters. Applying both of these tools to the zebrafish stool microbiome assemblies generated a set of putative MAGs. Next, we used the *bin_refinement* module of metaWRAP (Uritskiy et al., 2018) to perform refinement of putative MAGs. Briefly, refinement involved generating hybrid sets of putative MAGs from the two binning tools and identifying the best versions of each putative MAG from each group based on completeness and contamination estimates. The completeness and contamination were estimated using CheckM (v0.9.7) (Parks et al., 2015). Furthermore, the threshold for completeness and contamination were 50% and 20%, respectively. In addition, the *bin_refinement* module also ensured that one contig was not binned to more than MAG. Finally, for obtaining a final set of zebrafish MAGs, we removed MAGs with an overall MAG quality score (MQS) below 50. MQS was calculated as follows:


MQS =Completeness (%)−Contamination (%)

Based on MQS, we categorized the zebrafish MAGs into high quality (HQ; MQS ≥ 86), medium quality (MQ; MQS 71-85), low quality (LQ; 50-70). MAGs with MQS < 50 were discarded from further analyses.

### MAG Dereplication and Quantification

Next, we performed clustering and dereplication of the zebrafish MAGs to identify non-redundant MAGs in the dataset. To this end, we used dRep (v2.3.2) (Olm et al., 2017), which compares and clusters similar genomes by performing primary and secondary clustering. In dRep, we used Mash (Ondov et al., 2016) for performing primary clustering with an identity threshold of 99%. For secondary clustering, we used ANImf, which aligns the whole genomes with NUCmer (Marçais et al., 2018) and performs alignment filtration before comparing genomic regions, with an identity threshold of 95%. The threshold for minimum level of overlap between genomes for secondary clustering was set to 20% (options: ‘-pa 0.99, -sc 0.95, -nc 0.2, –S_algorithm ANImf’, –clusterAlg single). Lastly, the dereplicated representatives of each cluster were retrieved using *choose* module of dRep.

The quantification of MAGs was performed by determining the relative abundances of MAGs in our metagenome samples. This was achieved with CoverM (v0.5.0) (https://github.com/wwood/CoverM), which is specifically designed to calculate the relative abundance of genomes/MAGs from metagenomes. The *genome* module of CoverM was used with default parameters (except following parameters: –min-read-aligned-percent 0.75, –min-read-percent-identity 0.95, and –min-covered-fraction 0) for all-vs-all quantification i.e., all MAGs were quantified from all metagenomes.

### MAG Taxonomic and Functional Analysis

For taxonomic classification, we used the Genome taxonomy database toolkit (GTDB-Tk v1.7.0) (Chaumeil et al., 2019) and its current reference database (v202). Briefly, the *classify_wf* module of GTDB-Tk was used, which automatically performs the necessary steps required for taxonomic classification of MAGs (or genomes of interest). These include identifying marker genes from the MAG, generating multiple sequence alignment, and determining taxonomic classification for the MAGs. In addition, we also used GTDB-Tk for inferring the phylogenetic tree for the MAGs using the multiple sequence alignment (GTDB-Tk *infer*). The tree was visualized using the interactive tree of life (iTOL v6) webserver (Letunic and Bork, 2021).

The functional analysis was performed using Prokka (v1.14.0) (Seemann, 2014) and EnrichM (v0.6.4). The generalized annotation features (i.e., number of CDS, tRNAs) were obtained from Prokka using default parameters. EnrichM was used to identify the Kyoto Encyclopedia of Genes and Genomes (KEGG) orthology groups (KOs) (Kanehisa et al., 2014) in the MAGs (using EnrichM *annotate* module) to determine the metabolic pathways that the MAGs encodes by using KEGG modules as reference (using EnrichM *classify* module). EnrichM *classify* also provided the completeness levels of KEGG modules among the MAGs. KEGG modules were considered completely present in a MAG if all the required KOs were present in that MAG.

### Comparative Genomic Analysis of *Paucibacter* MAGs and Reference Genomes

For performing a comparative genomic analysis between the *Paucibacter* MAGs (identified in this study) and the reference genomes, we first downloaded the available *Paucibacter* genome sequences from NCBI GenBank (dated December 16, 2021). The Genus *Paucibacter* is represented by only three reference genomes and ten drafts. We further refer to these 13 genomes as *Paucibacter* reference genomes for convenience. The phylogenetic comparison between the 8 MAGs and 13 reference genomes involved identifying marker genes and their multiple sequence alignment, followed by subsequent inference of a phylogenetic tree using GTDB-Tk. The phylogenetic tree was visualized using the iTOL web server.

FastANI (v1.32) (Jain et al., 2018) was used (with default parameters) for computing whole-genome average nucleotide identity (ANI) between the *Paucibacter* MAGs and references. QUAST (v5.0.2) (Gurevich et al., 2013) was used to compare *Paucibacter* MAGs and references. EnrichM was used to identify KOs and determine the completeness of KEGG pathway modules. We also used the EnrichM *enrichment* module to determine the differentially enriched (DE) KOs between *Paucibacter* MAGs and references.

### Statistical Analysis

Most of the statistical analyses, including calculation of alpha diversity, linear regression, and group tests, were performed using the R (v4.0.3) programming language. Comparison between two groups was performed and tested using the non-parametric Wilcoxon test, and *P* < 0.05 was considered statistically significant. DE KOs were identified using EnrichM *enrichment* which also performed adjustment for multiple testing using Benjamini and Hochberg false discovery rate (FDR) controlling procedure. Adjusted *P* (*P_adj_*) < 0.25 was considered statistically significant.

## Results

### General Characteristics of the MAGs Recovered From Zebrafish Stool Microbiome

**Figure 1:**
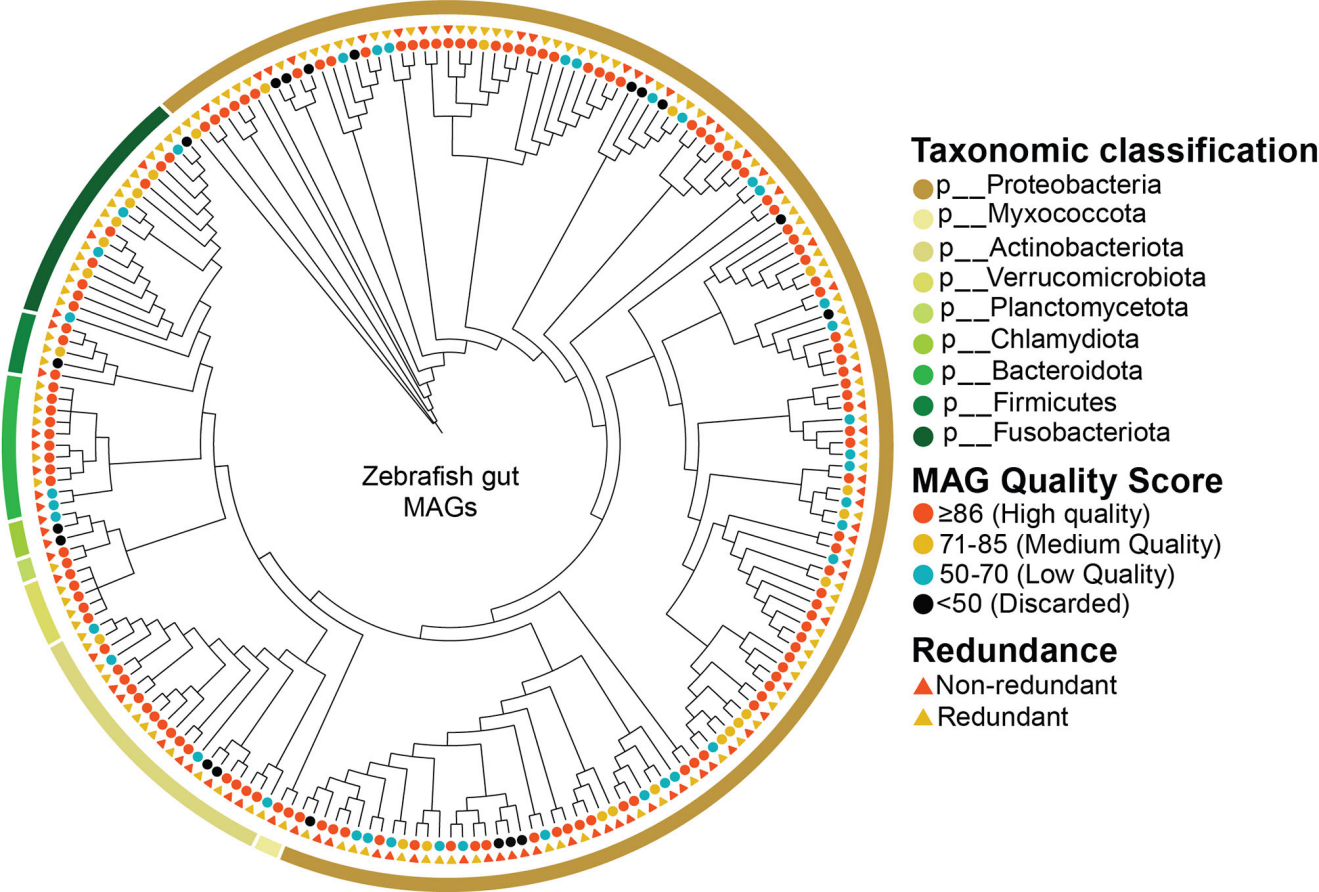
Taxonomic classification and general characteristics of MAGs identified from the zebrafish stool microbiome. The outer ribbon demonstrates the taxonomic classification (performed using GTDB-tk) of MAGs at the Phylum level, which shows that most MAGs belonged to the phylum Proteobacteria. MAG quality scores are shown through the circles, suggesting that most of the MAGs were High- or Medium Quality, whereas MAGs with scores less than 50 were discarded from further analysis. The quality scores were inferred from the MAGs completeness and contamination levels calculated using CheckM. Lastly, the redundancy between the zebrafish stool MAGs is shown using the triangles. MAGs were considered redundant if they shared more than 95% whole-genome ANI.

**Figure 2:**
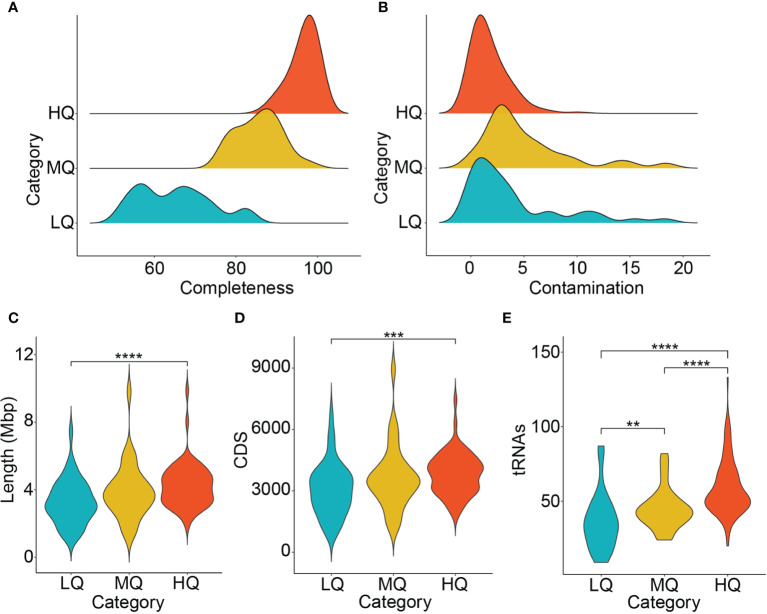
General characteristics of the zebrafish stool microbiome. **(A)** Completeness **(B)** Contamination **(C)** MAG length **(D)** Coding sequences (CDS) and **(E)** the number of predicted tRNAs in the zebrafish stool MAGs are shown. The comparison among MAG lengths, CDS, and tRNAs in different MAG groups was performed using the Wilcoxon test, and *P* < 0.05 was considered statistically significant. **P <= 0.01; ***P <= 0.001; ****P <= 0.0001.

Metagenome *de novo* assembly produced >320,000 contigs with a cumulative length of ~1.37 Gbps. The average N50 length was >11,000 bp, the average %GC was 56, whereas the largest identified contig had a size of 1.4 Mbp (**Supplementary Table 1**). The *de novo* assembly, genome binning, and refinement resulted in the recovery of more than 200 MAGs from the zebrafish stool microbiome (**Figure 1**). Categorizing the MAGs on MQS indicated that their vast majority were HQ (63%), with an MQS score of 94.9±4.07. Among these, 4 MAGs showed MQS of 100, which corresponds to 100% predicted completeness and 0% contamination (**Figures 2A, B**), whereas another 98 MAGs in this group showed MQS in the range of 90-99.5. The MQ group contained 32 MAGs with an MQS of 80.5±4.29, whereas in the category of LQ, the 39 MAGs had an MQS of 61.2±7.05. HQ and MQ groups collectively constituted ~80% of the total zebrafish stool MAGs, with an MQS of 91.9±7.15, suggesting that our multi-tool genome binning and refinement-based approach performed excellently (**Supplementary Table 2**).

In addition, we also determined the redundant and non-redundant (NR) fraction of zebrafish MAGs using dRep (as described in Materials and Methods), which showed that 73 of the MAGs (~38%) were NR. However, the downstream analyses are performed for the complete zebrafish MAG set.

The MAGs recovered from the zebrafish stool microbiome demonstrated an average genome size of 3.97±1.4 Mbp. The MAG lengths were significantly different between HQ and LQ groups (Wilcoxon test, *P* < 0.05), whereas no significant difference was observed between MAG lengths in HQ vs. MQ and MQ vs. LQ comparisons (Wilcoxon test, *P* > 0.05; **Figure 2C**). The HQ MAGs were contained in a minimum of 4 (MAG28) and a maximum of 928 (MAG33) contigs (**Supplementary Table 2**). In addition, the average number of CDS (~4000 per MAG) and predicted tRNAs (~60 per MAG) was also significantly higher in HQ MAGs than the MQ (~3900 CDS and 46 tRNAs) and LQ groups (~3200 CDS and 37 tRNAs; **Figures 2D, E**).

### The Taxonomic Landscape of the Zebrafish MAGs

Taxonomic classification of these MAGs using GTDB-Tk indicated that they belonged to 9 different phyla, including Proteobacteria, Actinobacteriota, Fusobacteriota, Bacteroidota, and others (**Figure 1** and **Supplementary Table 2**). The overwhelming majority of the zebrafish MAGs were classified to phylum Proteobacteria (~67%), with genomic representatives from 18 different families. These included Burkholderiaceae (29 MAGs), Enterobacteriaceae (18 MAGs), Aeromonadaceae (17 MAGs), Beijerinckiaceae (12 MAGs), Chromobacteriaceae (7 MAGs), and Rhodocyclaceae (7 MAGs) as the six most prevalent families. Phylum Actinobacteriota was represented by families Microbacteriaceae (14 MAGs), UBA8139 (3 MAGs), Mycobacteriaceae (2 MAGs), and CAIYMF01 (1 MAG). Fusobacteriota was represented by 18 MAGs, which all belonged to the family Fusobacteriaceae. In addition to the families from these phyla, members from several other families were also identified, listed in **Supplementary Table 2**.

**Figure 3:**
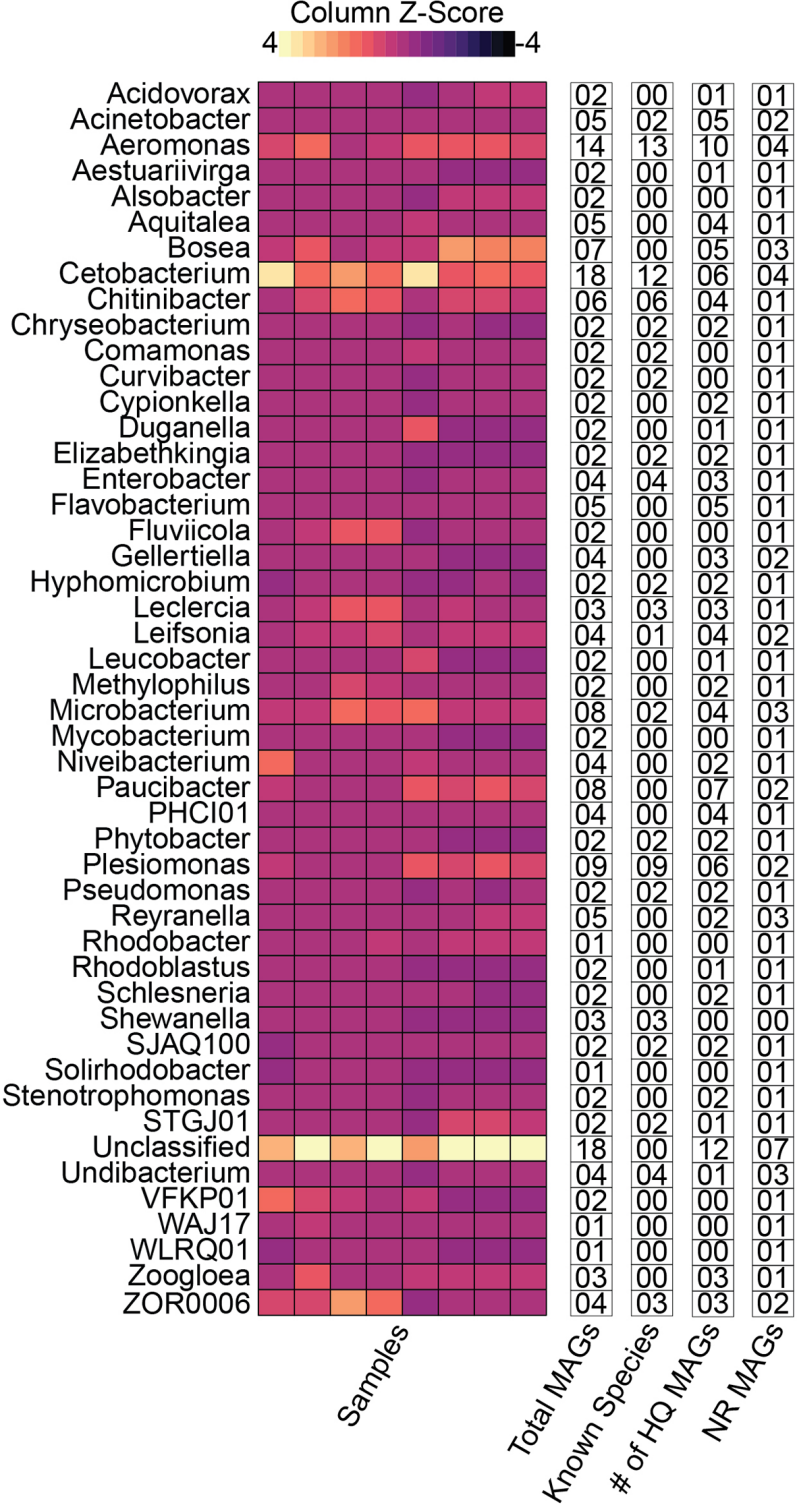
Distribution of the zebrafish MAGs at genus level and other categories. The relative abundance at the genus level is shown using the heatmap. The text columns highlight the following: (1) the total number of MAGs in a given genus, (2) the number of MAGs which could be successfully classified to species-level (3) the number of high-quality MAGs, and (4) the number of non-redundant (NR) MAGs in respective genera. These results indicate that majority of the MAGs were classified as members of *Cetobacterium* or an unclassified genus. In contrast, the highest number of MAGs (12) were successfully classified to species level from the genus *Aeromonas*. Additionally, the genus *Paucibacter* contained the highest number of unknown species relative to the total number of MAGs in the genus. In contrast, the Unclassified MAGs had the maximum number of NR MAGs.

At the genus level, zebrafish MAGs were classified into 47 different genera, among which, *Cetobacterium* (18 MAGs) and *Aeromonas* (14 MAGs) were most prevalent. In contrast, the lesser-known genera, *WAJ17* and *WLRQ01*, were represented by 1 MAG each. Quantification of MAGs (using relative abundance) also indicated that *Cetobacterium* had the highest relative abundance (>10%), followed by *Bosea* (5.48%), *ZOR0006* (4.9%), *Aeromonas* (4.76%), *Microbacterium* (4.74%), *Chitinibacter* (4.2%), *Leclercia* (3.18%), *Plesiomonas* (2.88%), *Paucibacter* (2.78%), and *Fluviicola* (2.59%). Among these genera, the highest number of HQ MAGs was contained in genus *Aeromonas* (10 MAGs), followed by *Paucibacter* (7 MAGs), *Cetobacterium* (6 MAGs), *Plesiomonas* (6 MAGs), and *Acinetobacter*, *Bosea*, and *Flavobacterium* (5 MAGs each). These results are demonstrated in **Figure 3**.

Zebrafish stool MAGs were classified into 23 different known species. According to the relative abundances, Cetobacterium somerae, ZOR0006 sp000798955, Chitinibacter tainanensis, Aeromonas hydrophila, Leclercia adecarboxylata, and Plesiomonas shigelloides were highly abundant. In contrast, Plesiomonas shigelloides (9 MAGs), Aeromonas jandaei (7 MAGs), Cetobacterium somerae (6 MAGs), Chitinibacter tainanensis (6 MAGs), and Aeromonas hydrophila (5 MAGs) were most prevalent (**Supplementary Table 3**).

### Unexplored Microbial Diversity Dominated the Zebrafish Stool Microbiome

Taxonomically, the vast majority of the zebrafish stool MAGs (~99%) were successfully classified at the family level. However, beyond the family level (i.e., at genus and species levels), most of these MAGs remained unclassified and suggested the presence of previously unexplored and potentially novel microbial species in the zebrafish stool microbiome. In total, 113 MAGs (~60%) could not be classified as any known species using GTDB-Tk and its most recent taxonomic database (released April 23, 2021). The most prominent examples, in this case, include MAGs belonging to the genus *Aquitalea*, *Bosea*, *Flavobacterium*, *Paucibacter*, and *Reyranella*. Collectively, these five genera consisted of 30 MAGs, none of which could be successfully classified into a species (**Figure 3**). *Paucibacter* had the highest number of MAGs (8), followed by *Aquitalea* (7). GTDB-Tk infers taxonomy using the topological placement of MAGs in the reference genome tree and through whole-genome average nucleotide identity (ANI) with the reference genomes. Hence, these MAGs could not be classified into known species through both approaches. ANI could only be computed for certain MAGs lower than the threshold required for genomes to be considered the same species (ANI <95%). These included six *Paucibacter* MAGs (MAG2, MAG36, MAG58, MAG144, MAG174, and MAG178), and four *Reyranella* MAGs (MAG61, MAG95, MAG166, and MAG198), and two *Bosea* MAGs (MAG50, MAG51).

Genus *Bosea* and *Reyranella* contained three NR MAGs each, whereas *Cetobacterium*, *Gellertiella*, *Microbacterium*, and *Paucibacter* contained two NR MAGs. In addition to these genera, 28 other NR MAGs remained unclassifiable at the species level (**Figure 3**). These unclassified NR MAGs represent potentially novel species within their corresponding genera.

### Zebrafish Stool MAGs Were Functionally Highly Diverse

**Figure 4:**
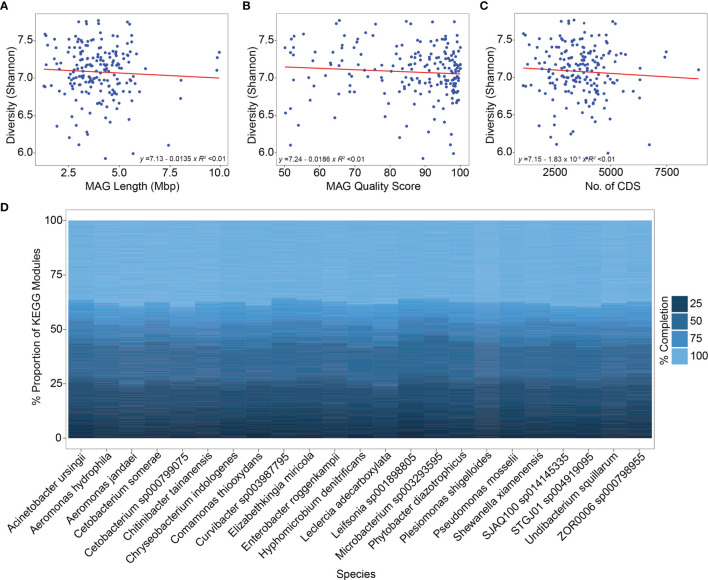
Metabolic diversity-MAG characteristics’ association and distribution of KEGG pathway modules in different species. **(A–C)** The correlation between metabolic diversity (inferred using the Shannon index), MAG Length, MAG quality score, and the number of predicted CDS is shown. Linear regression was performed to determine these correlations with the linear fitting model. The results indicate no significant association between metabolic diversity and the three mentioned parameters (*R^2^* < 0.01). **(D)** The relative proportion of different KEGG metabolic pathway modules is shown among the species that were successfully classified from the zebrafish stool MAGs. Nearly 40% of the metabolic pathway modules demonstrated a completion level of 100% whereas the remaining showed completion levels between 25-75%.

Next, we sought to determine the functional (metabolic) potential of the zebrafish stool MAGs. We identified more than 6,200 different KOs (**Supplementary Table 4**) from the MAGs, which corresponded to >500 KEGG metabolic pathway modules (KMMs). The metabolic diversity (Shannon and Simpson indices) was comparable among the major phyla of the zebrafish stool microbiome (data not shown). We also determined the possible association between metabolic diversity and MAG length, MQS, and the number of CDS. However, there was no significant association between metabolic diversity and the three MAG parameters (**Figures 4A–C**).

Furthermore, the level of completion of the KMMs in the zebrafish stool MAGs was determined. A module was considered 100% complete only if all the required KOs for that module were identified from a MAG. This resulted in 475, 422, 354, and 338 KMMs with completeness levels of 25%, 50%, 75%, and 100%, respectively (**Supplementary Table 5**). Thus, nearly 70% of the KMMs were fully complete in the zebrafish stool MAGs, whereas ~84% of the total modules were at least 50% complete. The highly abundant species include *Cetobacterium somerae*, *ZOR0006 sp000798955*, *Chitinibacter tainanensis*, *Aeromonas hydrophila*, *Leclercia adecarboxylata*, and *Plesiomonas shigelloides*, ~40% of the KMMs were 100% complete. In addition, certain other species (for example, *Cetobacterium sp000799075* and *Comamonas thiooxydans*) also exhibited similar KMM completeness levels (**Figure 4D**).

**Figure 5:**
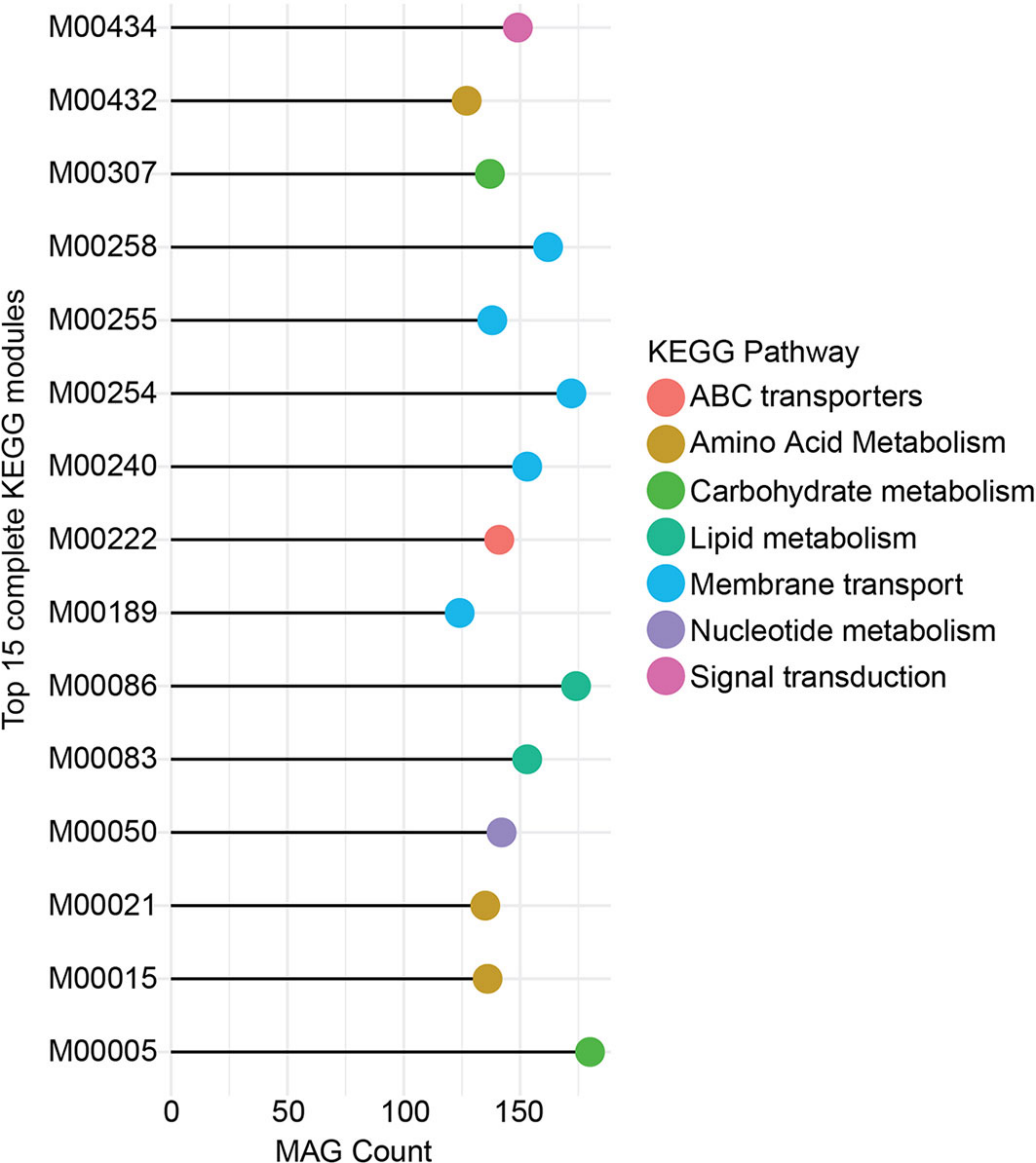
Top 15 complete KEGG pathway modules. Here, the 15 most complete pathway modules and the count of MAGs (in which they are identified to be 100% complete) are given. The color key indicates the association of the pathway module with the KEGG metabolic pathway categories. Module M00005 was identified to be 100% complete from more than 150 MAGs and belonged to the Carbohydrate metabolism category of KEGG pathways. M00086, M00254, and M00258 were the three other most prevalent metabolic modules.

Next, we determined the top 15 100% complete KMMs (based on prevalence) across the zebrafish stool MAG catalog. The KEGG module *M00005* (involved in Carbohydrate metabolism) was present in >150 MAGs. Similarly, *M00086*, *M00083* (Lipid metabolism), and *M00254* (Membrane transport) were also identified from more than 150 MAGs. Other KMMs corresponded to pathways involving amino acid metabolism, nucleotide metabolism, and signal transduction (**Figure 5**). These results suggest that the zebrafish stool MAGs, including highly abundant and less abundant members, are metabolically highly diverse and capable of performing various routine microbial metabolic processes.

### Comparative Analysis of *Paucibacter* MAGs and References

**Figure 6:**
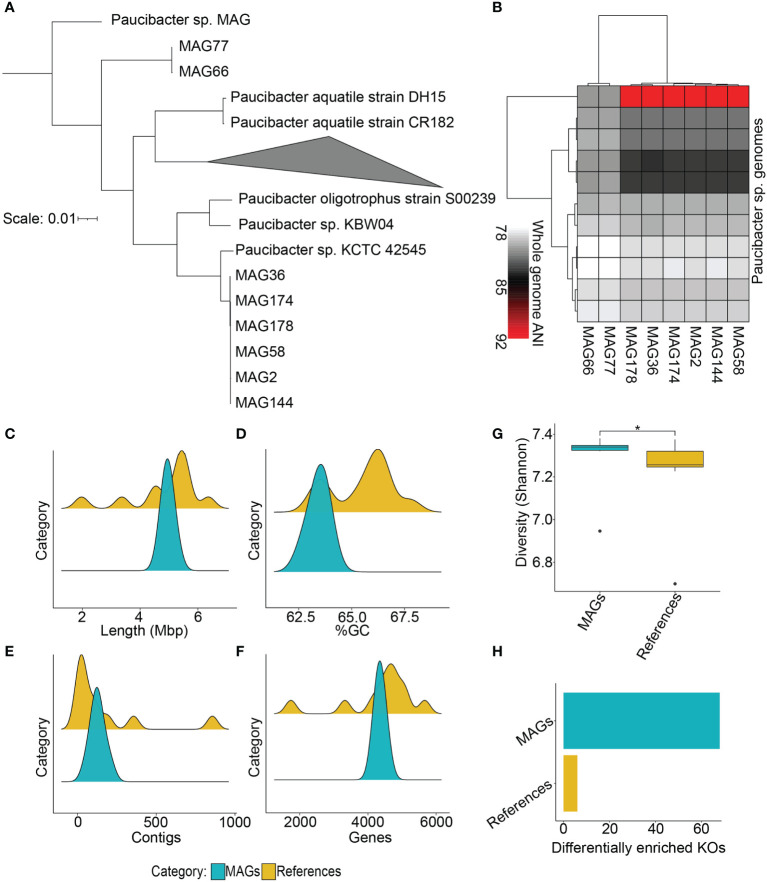
Comparative analysis of *Paucibacter* MAGs and reference genomes. **(A)** Phylogenetic analysis between *Paucibacter* MAGs and reference genomes is shown which indicates that the majority of MAGs were phylogenetically distinct from the reference species, as the MAGs clustered separately from them. **(B)** Whole-genome-based average nucleotide identity (ANI) analysis indicated that the majority of the *Paucibacter* MAGs shared at most 92% ANI with the reference genomes, indicating their potential of being novel species of the *Paucibacter* genus. **(C–F)** Comparisons of genome lengths, %GC, number of contigs, and number of predicted genes between MAGs and references. **(G)** Comparison of metabolic diversity (Shannon index) between the two groups indicates that MAGs had significantly higher diversity than references (Wilcoxon test, *P* < 0.05). **(H)** Barplot indicating the number of DE KOs in the two groups. The *Paucibacter* MAGs had a significantly higher number of DE KOs in contrast with *Paucibacter* reference genomes. *P <= 0.05.

Lastly, we compared the *Paucibacter* MAGs (identified from the zebrafish stool microbiome) and Paucibacter references (downloaded from the NCBI GenBank database) to highlight their similarities and differences. Phylogenetic analysis between the two groups demonstrated that the *Paucibacter* MAGs differed from the reference genomes. *Paucibacter* MAGs formed two distinct clusters, i.e., Cluster 1 and 2. Cluster 1 included MAG77 and MAG66, which appeared to be more distantly related to the remaining MAGs and references. These two MAGs were placed closest to a previously reported *Paucibacter* MAG (GenBank assembly accession: GCA 018780415.1). Cluster 2 included MAG2, MAG36, MAG58, MAG144, MAG174, and MAG178, which clustered closer to *Paucibacter* sp. KCTC 42545 (**Figure 6A**).

Pairwise ANI also confirmed the clustering pattern obtained from phylogenetic comparison, as it also produced two distinct clusters for the MAGs. In addition, from the pairwise ANI calculations, it became further evident that these 8 MAGs belong to a newer species within genus *Paucibacter* since their ANI with the reference genomes was <95% (**Figure 6B**), which does not satisfy the criteria to be considered the same species. We also compared several genomic features (total length, %GC, No. of contigs, and No. of genes) between the MAGs and reference genomes. Comparison of these features indicated that the total length, number of contigs, and number of predicted genes in the two groups were very similar, whereas %GC showed a slight difference (**Figures 6C–F**; **Supplementary Table 6**).

Finally, we compared the metabolic diversity and tested KOs’ differential enrichment (DE) between the *Paucibacter* MAGs and references. The results showed that the metabolic diversity (Shannon index) was significantly high in the MAGs in contrast with the reference genomes (Wilcoxon test, *P* < 0.05; **Figure 6G**). From testing the differential enrichment of KOs between the two groups, we identified 68 KOs, which showed DE in the MAGs (**Supplementary Table 7**), and only 6 KOs, which showed DE in the reference genomes (*P_adj_* < 0.25; **Figure 6H** and **Table 1**). Altogether, the comparative analysis highlighted the presence of taxonomic novelty and increased functional diversity in the *Paucibacter* MAGs recovered from the zebrafish stool microbiome in contrast with the currently available *Paucibacter* reference genomes.

**Table 1:** List of 10 most significantly enriched KOs in the *Paucibacter* MAGs and reference genomes.

KO ID	Enriched in	*P* value	*P_adj_* value	Function/Description
K03312	MAGs	0.0001	0.06	*gltS*; glutamate:Na+ symporter, ESS family
K09822	MAGs	0.0001	0.06	K09822; uncharacterized protein
K06871	MAGs	0.0001	0.06	K06871; uncharacterized protein
K02196	MAGs	0.0001	0.06	*ccmD*; heme exporter protein D
K05577	MAGs	0.0001	0.06	*ndhF*; NAD(P)H-quinone oxidoreductase subunit 5 [EC:7.1.1.2]
K07705	References	0.0033	0.19	*lytT*, *lytR*; two-component system, LytTR family, response regulator LytT
K07165	References	0.0033	0.19	*fecR*; transmembrane sensor
K12373	References	0.0033	0.19	HEXA_B; hexosaminidase [EC:3.2.1.52]
K01679	References	0.0033	0.19	E4.2.1.2B, *fumC*, FH; fumarate hydratase, class II [EC:4.2.1.2]
K06891	References	0.0062	0.24	*clpS*; ATP-dependent Clp protease adaptor protein ClpS

## Discussion

In this study, we performed the initial characterization of the zebrafish stool microbiome using genome-resolved metagenome analysis. We generated the first *de novo* assembly of adult zebrafish stool microbiome and recovered ~200 MAGs through genome binning and subsequent refinement. Although Gaulke and colleagues have also performed metagenome assembly and recovery of metagenome-assembled genomes from the zebrafish stool microbiome for generating an integrated gene catalog, their work is currently only available as a preprint (Gaulke et al., 2020). Therefore, to the best of our knowledge, the MAGs reconstructed in our study represent the first published catalog of microbial genomes recovered from the zebrafish stool microbiome. The overwhelming majority of these MAGs have a high-quality score (high completeness and low contamination level). At the same time, a significant proportion of these MAGs represents potentially novel species of their genera.

The structure of the zebrafish stool microbiome, deciphered by 16S rRNA and MGS sequencing, suggests that most of the microbiome in zebrafish is typically dominated by members of Proteobacteria, Actinobacteriota, Bacteroidota, Fusobacteriota, and ﻿Verrucomicrobiota (Roeselers et al., 2011; Kayani et al., 2021b). The MAGs recovered in this study predominantly belong to these phyla, while several members from Myxococcota, Planctomycetota, and Chlamydiota were also identified. Hence, this catalog of MAGs can be considered the first genomic representation of key microbial players in the zebrafish stool microbiome. The actual number of representative genomes in the presented catalog may be fewer since dereplication produced a set of 73 NR MAGs. The complete MAG catalog encompasses strain-level diversity from the zebrafish stool microbiome since we clustered the MAGs using a 95% similarity cutoff. The majority of the published datasets also contain a significant fraction of MAGs that share >99% similarity (Parks et al., 2017; Almeida A. et al., 2019; Pasolli et al., 2019). Maintaining such highly similar genomes in the total dataset could be extremely important, e.g., in explaining strain-level diversity, single-nucleotide variations, and variability in the auxiliary genes (present in different MAGs) of the same species (Evans and Denef, 2020) recovered (for example) from case-control microbiomes.

Our results demonstrate that *Cetobacterium somerae*, a member of the phylum Fusobacteriota, was the most abundant species (based on relative abundance) in the zebrafish stool microbiome. *C. somerae* was initially cultured from human feces (Finegold et al., 2003), but its presence has also been reported in different types of fish, including zebrafish (Tsuchiya et al., 2008; Roeselers et al., 2011; Larsen et al., 2014; Ray et al., 2017; Burgos et al., 2018). *C. somerae*, a Gram-negative and rod-shaped bacterium, has been reported to produce cobalamin (vitamin B_12_) and is suggested to be involved in determining the vitamin B12 requirements in freshwater fish (Roeselers et al., 2011). *Aeromonas hydrophila* is a Gram-negative, facultative anaerobe, a motile bacterium frequently identified in freshwater and sewage. *A. hydrophila* is often considered pathogenic for the fish since it may cause certain diseases in the fish (Abdelhamed et al., 2017). However, its nature as a primary pathogen of fish remains debatable since its infection in fish is usually observed due to extreme stress, physical trauma, and infection from another pathogen (Bruno and Ellis, 1996). Our previous study, which involved using MGS, showed that the healthy zebrafish stool microbiome contains *A. hydrophila* (Kayani et al., 2021b). Therefore, we argue that *A. hydrophila* is an essential member of the healthy zebrafish stool microbiome and should not be only associated with diseases in fish. However, their exact role in the zebrafish stool microbiome remains to be elucidated, requiring effort from the zebrafish researchers’ community.

The metabolic activities of the gut microbiome are highly essential for the host as they are known to provide several types of metabolites bioactive molecules and regulate the host’s immune system and metabolic pathways (Vernocchi et al., 2020). Due to its importance, the gut microbiome is often referred to as a virtual “metabolic organ” of the host (O'hara and Shanahan, 2006). The majority of the annotated genes from the zebrafish stool microbiome are involved in metabolism-associated pathways that include carbohydrate metabolism, nucleotide metabolism, amino acid metabolism, and lipid metabolism (Almeida A.R. et al., 2019; Kayani et al., 2021b). The MAGs recovered in this study showed high diversity (6000 different KOs) and affiliation with a wide range of metabolic functions (>500 KMMs) that showed high completeness level (338 KMMs were 100% complete). Interestingly, we did not find metabolic diversity significantly associated with the MAG length, MQS, and number of CDS. This suggests the presence of diverse metabolic functions even in smaller-sized MAGs. These KMMs were associated with similar metabolic pathways that have been reported from the zebrafish stool microbiome from the 16S rRNA- or MGS-based surveys (Almeida A.R. et al., 2019; Kayani et al., 2021b). The highly prevalent KMMs were mostly affiliated with carbohydrate metabolism, lipid metabolism, membrane transport, and nucleotide metabolism. Therefore, it is plausible that the zebrafish stool microbiome can satisfy its energy requirements and other metabolic processes through diverse mechanisms.

Previously unexplored and less commonly characterized microbial species accounted for a major proportion of the recovered MAGs. For instance, *Chitinibacter tainanensis*, *Leclercia adecarboxylata*, and *Comamonas thiooxydans* have not been previously reported from the zebrafish stool microbiome. In addition, the overwhelming majority of the MAGs remained unclassified at the species level. The catalog of zebrafish MAGs encompassed 47 different genera with only 80 successfully assigned a species label. GTDB-Tk also computes ANI in an attempt to classify genomes to species level if they satisfy the ANI threshold. However, apparently, there was not enough ANI between the unclassified MAGs with any of the ~260,000 reference genomes in the GTDB-Tk database. This is reflective of the extent of unexplored microbial sequences in the zebrafish stool microbiome and highlights how understudied the gut microbiome of zebrafish still remains. In our opinion, comparative genomic and phylogenetic analysis can provide key insights into the characteristics of these MAGs and assist in the identification of truly novel microbial species that can expand the current reference genome databases.

Among other genera in the zebrafish stool microbiome, *Paucibacter* was represented by 8 MAGs, all of which could not be classified as any of the *Paucibacter* species. The genus *Paucibacter* is currently represented by only three reference genomes and ten drafts (collectively called *Paucibacter* reference genomes for convenience in this study) in the NCBI GenBank (Schoch et al., 2020). In the phylogenetic tree, *Paucibacter* MAGs are mostly clustered separately. ANI of 95% and above is usually considered the optimal threshold for assigning genomes to the same species. In the case of *Paucibacter* MAGs and references, this threshold is not satisfied (Goris et al., 2007). Therefore, these observations prove that these MAGs should be considered novel species. However, only MAG77 and MAG174 were NR from the 8 *Paucibacter* MAGs. Therefore, these two MAGs could be regarded as two novel species in the genus *Paucibacter*, and a suitable nomenclature could be proposed. However, here we have not presented an appropriate nomenclature for the novel *Paucibacter* species but intent to do it after further careful analyses of these genomes in the near future.

This study has several limitations. Firstly, in the current study, the fecal samples from the zebrafish were collected from the tank water which can make it difficult to assess if the microbiome composition of the collected sample is only due to gut colonization or has also been affected by the tank environment. Although we moved zebrafish to smaller tanks with clean water, still the possibility of contamination of the fecal samples cannot be fully ruled out. This can only be elucidated through the introduction of a water-only control among the samples. Unfortunately, in this study, we did not introduce such control but we will add it in our future study designs to address this question. Secondly, our study is based on the MGS of fecal samples, which introduces a certain degree of bias in the results and may not completely depict the gastrointestinal (GI) microbiome (Donaldson et al., 2016; Tang et al., 2020). Analyzing samples from the GI tract (e.g. biopsies) in combination with fecal samples can overcome the bias introduced by analyzing only the fecal samples. Therefore, a study in the near future should be designed that includes both of the two sample types for accurate resolution of the zebrafish microbiome. Lastly, this study is based on a limited number of samples and should only be considered as a preliminary genome-resolved characterization of the zebrafish stool microbiome. Further studies, involving a larger pool of samples can greatly expand the overall quality and length of *de novo* assembly that will be instrumental in recovering a greater number of representative microbial genomes from the zebrafish stool microbiome.

## Conclusions

In summary, we report the first *de novo* assembly and the first set of MAGs from the zebrafish stool microbiome. These MAGs correspond to the core microbial taxa, which are known to exist in the zebrafish stool microbiome. An overwhelming majority of the MAGs have above-average quality scores, diverse metabolic potential, and no genome representative in the current reference genome sequence database. Together, these results expand our understanding of the structure and function of the zebrafish stool microbiome and provide a framework for designing comparative genomic and evolutionary studies in the future.

## Data Availability Statement

The raw metagenomic datasets described in the study have been submitted to the NCBI SRA database and could be retrieved using the following accession number: PRJNA806371, SRR12456161, SRR12456162, and SRR12456170. The de novo assemblies and the recovered MAGs are available for download from Figshare via the following DOIs: 10.6084/m9.figshare.19146539 and 10.6084/m9.figshare.19149572, respectively.

## Author Contributions

Conceptualization, MRK; Data curation, MRK, SZ, RF, KY and YQ; Formal analysis, MRK, SZ, and RF; Funding acquisition, LH; Investigation, LH; Methodology, MRK, KY, YQ and XY; Project administration, XY and LH; Resources, XY and LH; Supervision, LC and LH; Writing – original draft, MRK, SZ, RF, KY, YQ, and LH; Writing – review & editing, MRK, KY, YQ, and LH. All authors contributed to the article and approved the submitted version.

## Funding

This work was supported by the National Natural Science Foundation of China [No. 81874265], National Natural Science Foundation of China [No. 82073561], Shanghai Science and Technology Commission [No. 18411966600], Shanghai Science and Technology Commission [No. 19410740800], Shanghai Jiao Tong University School of Medicine [No. 2020002], Discipline Construction Plan from Shanghai Municipal Health Commission [GWV-10.1-XK01].

## Conflict of Interest

The authors declare that the research was conducted in the absence of any commercial or financial relationships that could be construed as a potential conflict of interest.

## Publisher’s Note

All claims expressed in this article are solely those of the authors and do not necessarily represent those of their affiliated organizations, or those of the publisher, the editors and the reviewers. Any product that may be evaluated in this article, or claim that may be made by its manufacturer, is not guaranteed or endorsed by the publisher.

## Abbreviations

MGS, Whole metagenome shotgun sequencing; MAGs, Metagenome-assembled genomes; QC, Quality control; MQS, MAG quality score; HQ, High quality; MQ, Medium quality; LQ, Low quality; GTDB-Tk, Genome taxonomy database toolkit; KEGG, Kyoto Encyclopedia of Genes and Genomes; KO, KEGG orthology groups; DE, Differentially enriched.
